# Supplementary material for: Harambee! 2.0: Community resources and resilience factors to leverage for improving HIV testing behaviors among African immigrant communities in Seattle, Washington
Source: PLoS One. 2025 Sep 19;20(9):e0331915. doi: 10.1371/journal.pone.0331915 (PMC12449009; doi:10.1371/journal.pone.0331915)
Supplement: S1 Supplementary Text — (DOCX) [file pone.0331915.s001.docx]

**Supplementary Text**

**Harambee 2.0! Key Informant Interview Guide**

“Thank you for talking with me today. Let’s go through the consent process first. [Once process is completed,] Do you have any questions? Ok, let’s get started. First, I would like to ask you some questions about your experiences with the healthcare system in the US.”

1. **Interviewee’s interactions with healthcare in US**

What has your experience been like interacting with the healthcare system in the US? Have you seen a physician or gone to a hospital in the US?

[If providers, ask about the scope of their work and interactions with African immigrant communities in Seattle…]

What barriers do people in your community face in accessing healthcare in the Seattle area?

- Providers- not from community, not culturally aware
- Insurance- difficulty getting insurance, confusion about how to use insurance
- Making appointments or finding doctors
- Interpreters, language, cultural differences
- Fear of impact on eligibility for longer term visa

1. **Barriers to health screenings**

What comes to your mind when I mention the words “health screenings”?

- What types of health screenings do people in your community have access to?
- What types of health screenings have you had done in your life? Or people you know have done?

[“Some people define health screenings as tests that are done by medical providers to identify health problems, ideally before someone has symptoms or feels ill because of the health problem. You have already mentioned some types of health screenings like mammograms for breast cancer or colonoscopy for colon cancer.”]

What are some issues around health screenings in your community in Seattle area?

- Do people from your community and people born in the U.S. use healthcare differently?
  - Why might one group be more likely to have health screenings?
  - Do some health screenings vary by sex or gender?

What are some things that keep people in your community in Seattle area from getting health screenings? What about, for example, blood pressure checks? Or mammograms for breast cancer detection? Or having annual physical exams?

1. **Stigma**

What does the word “stigma” mean to you?

- What types of stigma do people in your community experience?
- What types of stigma have you experienced before?

[“Some people define stigma as a set of negative beliefs that a society, group of people, or an individual has a about something. For example, people who have a mental illness may be viewed negatively by others because mental illness is stigmatized in their community.”]

Is there stigma around other specific health screenings, for example breast cancer screening?

- How does that stigma vary by condition, for example from breast cancer to colon cancer?
- Why might certain health conditions be stigmatized? What causes the stigma?
- Which specific health conditions do you think are stigmatized in this community? And why?

“Now let’s talk more about HIV… HIV is the virus that causes AIDS. Like many other illnesses, identifying HIV early and starting treatment can help a person with HIV to stay healthy.”

How does stigma affect HIV?

- What are the different types of stigma that affect HIV?
  - What causes the stigma surrounding HIV?
  - How does your community view people living with HIV? Do people living with HIV in your community have negative feelings about themselves because of their HIV status?
    - - Shame, embarrassment, fear
- How are people who are living with HIV treated differently than those without HIV by people in your community?
  - - Discrimination, prejudice, isolation
- How do healthcare providers or systems treat people living with HIV differently?
- What issues at the society-level might influence stigma against HIV?

Now, specifically focusing on testing for HIV, how does stigma affect HIV testing?

- What drives the stigma surrounding HIV testing?
- What are some additional barriers around testing for HIV?
- How do feelings about people living with HIV affect people’s willingness to be tested for HIV in your community?
- How do healthcare providers or systems worsen any stigma around HIV testing?
- What issues at the society-level affect stigma around HIV testing?

1. **Intersectional stigmas that my affect testing for HIV**

“People may experience other stigmas because of their behaviors or parts of their identity. For example, drug use may be stigmatized. Sometimes, people might be dealing with multiple stigmas, such as someone who injects drugs and is also HIV-positive.”

Can you think of other stigmas that may keep people from testing for HIV in your community?

- Immigrants
- Gay men, homosexuality, men who have sex with men (?)
- Injection drug use
- People who exchange sex
- Mental illness
- Racial, ethnic, religious, linguistic minorities

Are there things that are at the society level that contribute to these stigmas?

- Laws that make drug use illegal
- Immigration laws, refugee status
- Residential segregation

What are the society-level results of some of the stigmas that we talked about?

- Laws that criminalize HIV

1. **Decreasing stigma around health screenings**

What are some ways to increase HIV and other health screenings in your community?

If you were put in charge of planning a project to increase HIV testing in your community, what would you do?

- How do you think that project should be delivered?
- Where should the project take place?
- Who should be leading the project? Be implementing it?
- Who would be the best audience for that project?
- What format would you use?
  - - Possible examples: Video, education/workshops, religious sermons, public campaign
- What kinds of stigma would be important to address in this project?

**Harambee 2.0! Focus Group Discussion Guide**

**Facilitator/Moderator Introduction**

Hello, my name is _______________. I will be the moderator for the focus group discussion today. The purpose of today’s discussion is to hear your perspectives on Stigma and use of healthcare. My responsibility is to guide the discussion and make sure that everyone has an opportunity to speak and that no one dominates the conversation.

I am conducting this research on behalf of University of Washington in partnership with Somali/Ethiopian/Eritrean community.

READ THE CONSENT FORM AND ASK IF ANYONE HAS QUESTION

Before we go further, let us talk about some ground rules:

- There are no right or wrong answers; we’re interested in your honest ideas and opinions. We want to know YOUR thoughts, not what you think others will think, or what you think others want to hear.
- Our conversation is totally anonymous. We will not use your names in any report. However, we cannot guarantee the confidentiality of information among the group. Please don’t share your personal confidentially information or that of another.
- Our conversation today is being recorded for notetaking purposes. These recordings allow us to write a more complete report, and to make sure we accurately reflect your opinions. Please only speak one at a time, so that the recorder can pick up all your comments.
- Please turn off cell phones.
- At the end, we will give you $50 for your participation.

We will be asking a few questions about your thoughts about stigma in your community. Please feel to task any questions you may have beforehand and during the discussion. Everyone’s opinion is paramount, and we want to hear them.

**Possible Domains**

1. Stigma
   1. What does stigma mean to you?
   2. What types or kinds of stigma in healthcare exists in our community?
   3. What are the underlying factors that cause the stigma surrounding certain medical conditions?
   4. How does stigma in healthcare affect individual community members? (those stigmatized because of their conditions, for example, isolation, discrimination etc.)
   5. How do stigmatized illnesses affect individuals’ health seeking behavior? What about for HIV testing?
2. Intersectional stigma
   1. What are the multiple types of stigmas that affect our community? E.g. race or African, immigrant, gender/sex, unmarried/single, sexual orientation, religion.
   2. Immigrant status- what kinds of barriers do you face as belonging to an immigrant community? How do you think being an immigrant affects the other stigmas you have mentioned?
   3. How do multiple stigmas interact- race or African, gender/sex, unmarried/single? How do these multiple stigmas interact to influence HIV testing behavior?
   4. Would you say there is a hierarchy of the multiple stigmas in terms of impact?
   5. Any intergenerational differences, American-born vs. African/foreign-born?
3. Intervention
   1. Is there a role for religious communities or institutions in reducing HIV and other stigmas in our community?
   2. What type of project would you recommend to reduce HIV and other stigmas that affect HIV testing in your community?
   3. Present Project FAITH
      1. What role/s can religious leaders play in this project?
   4. What should our intervention look like?
      1. Who should be the audience?
      2. Who should deliver it?
      3. Format- how should it best be delivered?
      4. Location/timing- when and where should it be delivered?
      5. Topics- what topics or areas should be covered?
