# Supplementary material for: Harambee! 2.0: Community resources and resilience factors to leverage for improving HIV testing behaviors among African immigrant communities in Seattle, Washington
Source: PLoS One. 2025 Sep 19;20(9):e0331915. doi: 10.1371/journal.pone.0331915 (PMC12449009; doi:10.1371/journal.pone.0331915)
Supplement: Attachment — Submitted filename: Reviewer 1.docx [file pone.0331915.s005.docx]

**Harambee! 2.0: Community resources and resilience factors to leverage for improving HIV testing behaviors among African immigrant communities in Seattle, Washington**

This qualitative manuscript explores community resources and resilience factors that can be leveraged to improve HIV testing behaviors among African immigrant communities, specifically Ethiopian, Somali, and Eritrean, in Seattle, Washington. Through 30 key informant interviews (KIIs) and five focus group discussions (FGDs) with a total of 72 participants, the authors identified three key themes for intervention: (1) the critical role of religious leaders and institutions as facilitators, (2) the importance of leveraging existing community-based organizations and health professionals, and (3) the strategic use of culturally-rich and multilingual media to promote HIV education and testing. The authors are to be commended for centering a strengths-based, community-partnered approach, moving away from deficit-based narratives that have historically characterized public health interventions in immigrant communities. The manuscript contributes valuable insight into pathways for culturally congruent HIV stigma reduction and testing promotion within African immigrant contexts in the U.S. The depth of engagement with community partners, reflexivity on power and language dynamics, and incorporation of a social justice and health equity lens are significant strengths of this work

**Introduction**

- The introduction clearly sets out the rationale for the study and situates it within ongoing public health priorities (EHE).
- The pivot away from deficit-focused narratives is well articulated and timely.
- The authors provide strong epidemiological grounding specific to King County and African immigrants.
- The introduction could benefit from a slightly stronger framing of how this study builds on existing frameworks for reducing stigma and HIV prevention. A more direct reference to implementation science or community-engaged intervention literature would help position the novelty.
- The mention of Harambee! 1.0 is helpful; it might be worth adding a sentence clarifying how Harambee! 2.0 differs from or extends that work.

**Methods**

- The justification for using both KIIs and FGDs is well explained and appropriate.
- The attention to language, positionality, and consent procedures is commendable and demonstrates ethical rigor.
- The sampling strategy and community partnership approach are strengths of this work.
- Consider clarifying how divergent perspectives across KIIs and FGDs (or between PLWH and other groups) were systematically captured in analysis (beyond noting this in the Results). Were comparison queries or any analytic memos used in NVivo?
- The discussion of language (“preferred” vs. “dominant”) is very thoughtful but slightly long. Consider tightening to improve flow.

**Results**

- The results are well structured and thematically clear.
- The use of direct participant quotes is strong and gives voice to the communities.
- Table 2 is an excellent addition and provides good transparency.
- While the decision to combine KII and FGD data is justified, further explicit signposting of where key divergences occurred (especially between PLWH vs. religious leaders, or FGDs vs. KIIs) would enrich the depth of analysis.
- Some subthemes could be better separated. For example, the section on leveraging PLWH as educators could be elevated more clearly as a distinct theme or subtheme.
- Consider integrating a brief summary paragraph at the end of the *Results,* highlighting key points of agreement vs. divergence across communities and participant types.

**Discussion**

- The Discussion is significantly strengthened in this revision, well-structured, and now more clearly linked to prior literature.
- The section on applying a health equity and social justice lens is particularly well done.
- The implications for practice are thoughtful and actionable.
- There is still some residual repetition between the Results and Discussion. the first paragraphs under each subheading in the Discussion could be tightened.
- The contribution to broader HIV stigma reduction and implementation science literature could be more explicitly articulated. For example, how does this advance multilevel stigma reduction frameworks? How might this inform the adaptation of interventions for other immigrant groups?
- The limitations section is improved. Consider briefly mentioning potential social desirability bias in community-based FGDs on a sensitive topic like HIV.

**Conclusion**

- The Conclusion is clear and reinforces the study’s key messages.
- The focus on community assets and resilience is well aligned with the framing of the paper.
- Consider adding a forward-looking sentence about potential next steps (e.g., developing and piloting an intervention based on these findings, future partnerships, contribution to EHE goals).

This is an important, thoughtful, and well-executed manuscript. It advances both the empirical literature on HIV prevention among African immigrants in the U.S. and contributes to broader conversations on asset-based, community-engaged health equity research. The authors are to be commended for their responsiveness to prior reviews and their commitment to centering community voices. With minor revisions focused on clarity, flow, and a few methodological clarifications, this manuscript will be ready for publication. I look forward to seeing it in print.
