# Supplementary material for: Harambee! 2.0: Community resources and resilience factors to leverage for improving HIV testing behaviors among African immigrant communities in Seattle, Washington
Source: PLoS One. 2025 Sep 19;20(9):e0331915. doi: 10.1371/journal.pone.0331915 (PMC12449009; doi:10.1371/journal.pone.0331915)
Supplement: Attachment — Submitted filename: Reviewer Response 2025 07 27.docx [file pone.0331915.s007.docx]

**Reviewer 1 Comments and Responses: Word Document**

**1. Introduction Comments:**

- The introduction clearly sets out the rationale for the study and situates it within ongoing public health priorities (EHE). The pivot away from deficit-focused narratives is well articulated and timely. The authors provide strong epidemiological grounding specific to King County and African immigrants.
  **Response:** We thank the reviewer for this positive feedback. We are pleased that our introduction effectively situates the study within EHE priorities and that our shift toward strength-based rather than deficit-focused narratives was well-received. We appreciate the recognition of our locally-specific epidemiological grounding for King County and African immigrant communities.
- The introduction could benefit from a slightly stronger framing of how this study builds on existing frameworks for reducing stigma and HIV prevention. A more direct reference to implementation science or community-engaged intervention literature would help position the novelty.
  **Response:** We thank the reviewer for this constructive feedback. We agree that strengthening our theoretical grounding would enhance the introduction's impact. We have revised to more explicitly reference implementation science frameworks (such as the Consolidated Framework for Implementation Research) and community-engaged intervention literature that guides stigma reduction and HIV prevention efforts. We have also better articulated how our asset-based approach builds upon established community-engaged research principles, positioning our work's novel contribution within this broader scholarly context.
- The mention of Harambee! 1.0 is helpful; it might be worth adding a sentence clarifying how Harambee! 2.0 differs from or extends that work.
  **Response:** We thank the reviewer for this suggestion. We agree that clarifying the distinction between our two projects would be helpful for readers. We have add a sentence explaining that while Harambee! 1.0 identified stigma as a barrier through service delivery, Harambee! 2.0 extends this work by conducting in-depth qualitative exploration of stigma mechanisms while simultaneously identifying community assets and resilience factors using an explicitly strength-based framework.

**2. Methods Comments:**

- The justification for using both KIIs and FGDs is well explained and appropriate. The attention to language, positionality, and consent procedures is commendable and demonstrates ethical rigor. The sampling strategy and community partnership approach are strengths of this work.
  **Response:** We thank the reviewer for this positive feedback on our methodological approach. We are pleased that our rationale for combining KIIs and FGDs was clear and that our attention to language considerations, researcher positionality, and consent procedures was recognized. We appreciate the acknowledgment of our sampling strategy and community partnership approach, as these were central to ensuring culturally responsive and ethically sound research practices.
- Consider clarifying how divergent perspectives across KIIs and FGDs (or between PLWH and other groups) were systematically captured in analysis (beyond noting this in the Results). Were comparison queries or any analytic memos used in NVivo?
  **Response:** We thank the reviewer for this methodological clarification. We agree that our current description does not adequately detail how we systematically captured divergent perspectives across data collection methods and participant groups. We have revised the data analysis section to clarify that we used NVivo's comparison queries to systematically examine differences between KII and FGD responses, as well as between PLWH and other participant categories. Additionally, we maintained analytic memos throughout the coding process to document emerging patterns of convergence and divergence across these groups, which informed our thematic analysis meetings and helped ensure that minority or contradictory perspectives were not overlooked in our final themes.
- The discussion of language (“preferred” vs. “dominant”) is very thoughtful but slightly long. Consider tightening to improve flow.
  **Response:** We thank the reviewer for this feedback. We have condensed this section to maintain the key concepts while improving readability, focusing on the essential distinctions between "preferred language" (what participants are most comfortable using), "dominant language" (English as the language of power), and our intentional avoidance of "limited English proficiency" terminology that frames non-English preference as deficient.

**3. Results Comments:**

- The results are well structured and thematically clear. The use of direct participant quotes is strong and gives voice to the communities. Table 2 is an excellent addition and provides good transparency.
  **Response:** We thank the reviewer for this positive feedback on our results presentation. We are pleased that our thematic structure was clear and that the direct participant quotes effectively amplified community voices, this was a priority for us in ensuring authentic representation of participants' experiences. We also appreciate the recognition of Table 2's contribution to transparency, as we felt it was important to provide readers with clear insight into our analytical process and thematic development.
- While the decision to combine KII and FGD data is justified, further explicit signposting of where key divergences occurred (especially between PLWH vs. religious leaders, or FGDs vs. KIIs) would enrich the depth of analysis.
  **Response:** We thank the reviewer for this important observation. We have addressed this feedback by explicitly stating any key divergences throughout our revised Results section.
- Some subthemes could be better separated. For example, the section on leveraging PLWH as educators could be elevated more clearly as a distinct theme or subtheme.
  **Response:** We agree with this suggestion and have made necessary edits. The concept of leveraging PLWH as community educators represents a distinct and important finding that deserves greater prominence.
- Consider integrating a brief summary paragraph at the end of the *Results,* highlighting key points of agreement vs. divergence across communities and participant types.
  **Response:** We thank the reviewer for this excellent suggestion. We have added a summary paragraph at the end of the Results section that synthesizes the key points of convergence and divergence across the three communities and between participant types. This will provide readers with a clear analytical synthesis before moving to the Discussion.

**4. Discussion Comments:**

- The Discussion is significantly strengthened in this revision, well-structured, and now more clearly linked to prior literature. The section on applying a health equity and social justice lens is particularly well done. The implications for practice are thoughtful and actionable.
  **Response:** We thank the reviewer for this positive feedback on our revised Discussion section. We are pleased that the health equity and social justice framing was well-received and that our practice implications were found to be actionable.
- There is still some residual repetition between the Results and Discussion. the first paragraphs under each subheading in the Discussion could be tightened.
  **Response:** We agree that tightening the opening paragraphs of each Discussion subheading would improve flow and reduce redundancy. We have revised these sections to focus on interpretation and implications rather than restating findings, ensuring the Discussion adds analytical value beyond what was presented in the Results.
- The contribution to broader HIV stigma reduction and implementation science literature could be more explicitly articulated. For example, how does this advance multilevel stigma reduction frameworks? How might this inform the adaptation of interventions for other immigrant groups?
  **Response:** We thank the reviewer for this important suggestion. We have added a section to the discussion of how our findings advance multilevel stigma reduction frameworks, particularly the Earnshaw model we referenced, and how our asset-based approach could inform adaptation of interventions for other immigrant populations. We have also better articulated our contributions to implementation science literature regarding community-engaged approaches in stigmatized health conditions.
- The limitations section is improved. Consider briefly mentioning potential social desirability bias in community-based FGDs on a sensitive topic like HIV.
  **Response:** We thank the reviewer for this suggestion and have added a sentence to highlight this bias.

**5. Conclusion Comments:**

- The Conclusion is clear and reinforces the study’s key messages.
  **Response:** We thank the reviewer for this positive feedback. We are pleased that our conclusion effectively reinforces the study's key messages and provides clear synthesis of our findings.
- The focus on community assets and resilience is well aligned with the framing of the paper.
  **Response:** We appreciate this recognition of our consistent framing throughout the paper.
- Consider adding a forward-looking sentence about potential next steps (e.g., developing and piloting an intervention based on these findings, future partnerships, contribution to EHE goals).
  **Response:** We thank the reviewer for this excellent suggestion. We have added a forward-looking sentence to our conclusion that outlines our next steps, including plans to develop and pilot an intervention based on these findings in partnership with the same communities, and how this work will contribute to EHE goals in King County.
