# Supplementary material for: Harambee! 2.0: Community resources and resilience factors to leverage for improving HIV testing behaviors among African immigrant communities in Seattle, Washington
Source: PLoS One. 2025 Sep 19;20(9):e0331915. doi: 10.1371/journal.pone.0331915 (PMC12449009; doi:10.1371/journal.pone.0331915)
Supplement: Attachment — Submitted filename: PLOS ONE Reviewer comments.docx [file pone.0331915.s002.docx]

**Reviewer’s comments for Editors**

**Introduction**

The sentence: “Here, our qualitative findings further highlight--, state too early. I suggest move to the beginning of the Result section.

**Methods**

*Data Collection:*

The decision to use KII and FGDs need to be substantiated and located within other qualitative research methods.

*Sampling:*

What are the criteria for participants in KII and FGDs?

Could KII participants be part of the FGDs or not? And why or why not?

*Interviewers and facilitators:*

Did the authors not have their criteria for interviewers and FGD facilitators? Relying solely on community partners may come with biases like favoritism.

*Interview/discussion procedures:*

Is it community’s or group’s preferred language?

“The KIIs were conducted in several locations---. Interviewer and interviewee preference-“

I think Interviewee preference of location must supersedes that of the interviewer.

“Audio from KIIs and FGDs was recorded and then translated and transcribed into English.”

I suggest: We recorded the KIIs and FGDs verbatim and were translated and transcribed into English.

**Results**

We need to mentation Table 1 as reflecting the background/social characteristics of participants.

The authors seemed to combine the data of KII and FGDs. It would be clear to see the similarities and differences, particularly amongst the divergent individuals and differences.

How did religious leaders see themselves as gate keepers?

I think as a qualitative research paper, we need to see what the KII and FGDs participants said differently and differently. Using direct quotes will show the robustness of the findings and boost our understandings of the data.

Separation of KII and FGDs data/findings is paramount.

The authors need to show where the data stopped from their views.

Do PLWH views the same or differ from the of religious leaders and community members.

Be more explicit on how the COVID pandemic added to the differences between KII and FGDs’ data.

**Discussion**

Start the sections to reflect the first paragraph. Therefore, start with:

HIV education in religious organizations, followed by

Utilizing existing community resources, and finally

Developing and implementing an intervention.

Major findings must be linked to past studies in terms of were they are similar and different. This section further depicts the contribution to knowledge and new contribution to knowledge.

**Conclusion**

In conclusion, our findings show the significant challenges ----, which can be addressed --- in these studies communities, Ethiopian, Somali and Eritrean.

How would the pandemic define and shape the findings and health promotion activities in the communities. Would the approach be the same or differ in terms of placed preference.

What about stigma reduction amongst PLWH participants?
